# Preventing Thrombohemorrhagic Complications of Heparinized COVID-19 Patients Using Adjunctive Thromboelastography: A Retrospective Study

**DOI:** 10.3390/jcm10143097

**Published:** 2021-07-14

**Authors:** Connor M. Bunch, Anthony V. Thomas, John E. Stillson, Laura Gillespie, Rashid Z. Khan, Nuha Zackariya, Faadil Shariff, Mahmoud Al-Fadhl, Nicolas Mjaess, Peter D. Miller, Michael T. McCurdy, Daniel H. Fulkerson, Joseph B. Miller, Hau C. Kwaan, Ernest E. Moore, Hunter B. Moore, Matthew D. Neal, Peter L. Martin, Mark L. Kricheff, Mark M. Walsh

**Affiliations:** 1 Department of Internal Medicine, Indiana University School of Medicine South Bend Campus, Notre Dame, IN 46617, USA; cmbunch@iu.edu (C.M.B.); anvthoma@iu.edu (A.V.T.); jstills@iu.edu (J.E.S.); nzackari@iu.edu (N.Z.); 2 Department of Quality Assurance and Performance Improvement, Saint Joseph Regional Medical Center, Mishawaka, IN 46545, USA; gillesla@sjrmc.com; 3 Department of Hematology, Michiana Hematology Oncology, Mishawaka, IN 46545, USA; rkhan@mhopc.com; 4 Department of Internal Medicine, Boston University School of Medicine, Boston, MA 02118, USA; faadil.shariff@gmail.com; 5 Department of Internal Medicine, Saint Joseph Regional Medical Center, Mishawaka, IN 46545, USA; malfadhl@iu.edu (M.A.-F.); nmjaess@nd.edu (N.M.); 6 Department of Interventional Radiology, Saint Joseph Regional Medical Center, Mishawaka, IN 46545, USA; PMiller@xrcmi.com; 7 Division of Pulmonary and Critical Care, University of Maryland School of Medicine, Baltimore, MD 21201, USA; DrMcCurdy@gmail.com; 8 Department of Neurosurgery, Beacon Medical Group, South Bend, IN 46601, USA; dfulkers1@gmail.com; 9 Department of Emergency Medicine, Henry Ford Hospital, Detroit, MI 48202, USA; JMILLER6@hfhs.org; 10 Division of Hematology and Oncology, Department of Medicine, Northwestern University Feinberg School of Medicine, Chicago, IL 60611, USA; h-kwaan@northwestern.edu; 11 Department of Surgery, Ernest E. Moore Shock Trauma Center at Denver Health, Denver, CO 80204, USA; Ernest.moore@dhha.org (E.E.M.); hunter.moore@ucdenver.edu (H.B.M.); 12 Pittsburgh Trauma Research Center, University of Pittsburgh Medical Center, Pittsburgh, PA 15213, USA; nealm2@upmc.edu; 13 Department of Emergency Medicine, Louisiana State University Health Sciences Center, New Orleans, LA 70112, USA; pmart7@lsuhsc.edu; 14 Department of Emergency Medicine, Saint Joseph Regional Medical Center, Mishawaka, IN 46545, USA; Kricheff@gmail.com

**Keywords:** thromboelastography, anticoagulants, COVID-19, heparin, hemorrhage, coagulopathy, blood coagulation, blood coagulation tests, thrombosis

## Abstract

Background: The treatment of COVID-19 patients with heparin is not always effective in preventing thrombotic complications, but can also be associated with bleeding complications, suggesting a balanced approach to anticoagulation is needed. A prior pilot study supported that thromboelastography and conventional coagulation tests could predict hemorrhage in COVID-19 in patients treated with unfractionated heparin or enoxaparin, but did not evaluate the risk of thrombosis. Methods: This single-center, retrospective study included 79 severely ill COVID-19 patients anticoagulated with intermediate or therapeutic dose unfractionated heparin. Two stepwise logistic regression models were performed with bleeding or thrombosis as the dependent variable, and thromboelastography parameters and conventional coagulation tests as the independent variables. Results: Among all 79 patients, 12 (15.2%) had bleeding events, and 20 (25.3%) had thrombosis. Multivariate logistic regression analysis identified a prediction model for bleeding (adjusted R^2^ = 0.787, *p* < 0.001) comprised of increased reaction time (*p* = 0.016), decreased fibrinogen (*p* = 0.006), decreased D-dimer (*p* = 0.063), and increased activated partial thromboplastin time (*p* = 0.084). Multivariate analysis of thrombosis identified a weak prediction model (adjusted R^2^ = 0.348, *p* < 0.001) comprised of increased D-dimer (*p* < 0.001), decreased reaction time (*p* = 0.002), increased maximum amplitude (*p* < 0.001), and decreased alpha angle (*p* = 0.014). Adjunctive thromboelastography decreased the use of packed red cells (*p* = 0.031) and fresh frozen plasma (*p* < 0.001). Conclusions: Significantly, this study demonstrates the need for a precision-based titration strategy of anticoagulation for hospitalized COVID-19 patients. Since severely ill COVID-19 patients may switch between thrombotic or hemorrhagic phenotypes or express both simultaneously, institutions may reduce these complications by developing their own titration strategy using daily conventional coagulation tests with adjunctive thromboelastography.

## 1. Introduction

### 1.1. Background

Hypercoagulability has been well described in hospitalized patients with the severe acute respiratory syndrome coronavirus 2 (SARS-CoV-2) (i.e., the pathogen that causes the novel coronavirus disease 2019 (COVID-19)) [1,2,3]. Microvascular thrombosis is a ubiquitous finding in post-mortem examination of COVID-19 patients, even in the absence of macrovascular thrombosis [4,5,6]. The incidence of arterial and venous thrombi is particularly high in severely ill patients admitted to the intensive care unit (ICU), and thus, anticoagulating hospitalized COVID-19 patients remains paramount in their overall care [1,7,8,9]. Unfractionated heparin (UFH) may provide benefits beyond anticoagulation [10]. UFH possesses an anti-inflammatory effect at the endothelium, as well as the ability to interact with and bind the spike protein on COVID-19 viral particles [10]. In vivo models of coronavirus infection support the concept that UFH behaves as a “decoy receptor/sink to reduce viral infectivity and potentially augment viral clearance” [10,11]. Despite the frequent hypercoagulable state of hospitalized COVID-19 patients and the attractive therapeutic hypothesis of heparinoids, anticoagulation is often complicated by the rapid evolution from heparinoid resistance to heparinoid hypersensitivity, which may result in major hemorrhage [9,12,13,14,15,16]. With little evidence early in the pandemic, institutions offered conflicting guidelines for the empiric escalation of prophylactic anticoagulation to intermediate or therapeutic doses for hospitalized patients with COVID-19 pneumonitis and without macrovascular thrombosis [17,18,19,20,21].

### 1.2. Motivation

While evidence was actively evolving early in the pandemic, the observed high incidence of thrombohemorrhagic complications prompted our institution to establish a COVID-19-associated coagulopathy (CAC) committee. This CAC committee was inspired by an extracorporeal membrane oxygenation (ECMO) anticoagulation team that followed daily clinical changes, conventional coagulation tests (CCTs), anti-Xa levels, and viscoelastic tests [22,23]. In the ECMO literature, Colman et al. state, “guidelines recommend antithrombotic therapy during ECMO, but the guidelines leave it up to individual institutions to develop their own titration strategy” [22]. Given the unique nature of providing anticoagulation without evidence-based guidelines during a novel pandemic, we elected to establish a committee similar to that of ECMO, where there is a much longer history of using viscoelastic testing as part of the “titration strategy” [22].

Studying the adjunctive use of viscoelastic tests to guide anticoagulation was driven not only by the demonstrated use in ECMO, but also by early hematology society publications, which confronted the difficulty of practicing without the direction of evidence-based medicine. For example, in two authoritative commentaries, it was stated, “For clinicians trained in using an evidence-based medicine approach, we find ourselves forced to practice without data” [4], and, “On the other hand, it also offers opportunities: Comparison of treatment or prophylaxis management schemes that differ per center may offer an alternative for randomized clinical trials under certain conditions” [24].

Our CAC committee comprises a hematologist, a transfusion specialist, clinical pharmacists, and nursing staff, who together monitored the daily hemostatic phenotype of every COVID-19 inpatient and adjudicated anticoagulation. The CCTs (i.e., activated partial thromboplastin time (aPTT), prothrombin time (PT), fibrinogen, D-dimer, and platelet count) have not demonstrated prediction of bleeding risk in this group of patients [25]. Moreover, it has been demonstrated that aPTT can be falsely prolonged in COVID-19 patients at admission, and thus, may be an unreliable test for safely and effectively titrating heparin [26,27]. We hypothesized that for COVID-19 patients in the ICU—much like ECMO—focused attention by a diligent CAC committee using adjunctive thromboelastography (TEG) may reduce rates of bleeding and thromboses simultaneously. TEG and other viscoelastic tests have recently been used in small studies to diagnose and treat CAC [28].

### 1.3. Previous Work

This current study is an expanded analysis of a previously published pilot study [29]. In the previous study, we compared the ability of TEG parameters and CCTs to predict hemorrhage for anticoagulated COVID-19 patients in the ICU. Among the 10 bleeding and 21 non-bleeding patients, the results demonstrated significant differences on the day of bleeding for medians of the reaction time (R), clot kinetics (k), α-angle, PT, aPTT, and fibrinogen. In contrast, this study here analyzes the ability of TEG parameters and CCTs to predict hemorrhage among 12 bleeding and 67 non-bleeding patients by logistic regression modeling. The previous study comprised a heterogeneous group of patients confounded by patients anticoagulated with enoxaparin; in contrast here, this study comprises 79 patients treated exclusively with UFH. A second analysis from the pilot study also showed a significant decrease in bleeding incidence after adopting a non-bolus UFH dose guided by a TEG/CCT-algorithm. This study here builds upon the second analysis by also analyzing patient demographics and blood product usage after adopting the non-bolus UFH therapy and TEG/CCT-algorithm. Lastly, this study also details the frequency of macrovascular thromboses among the same 79 patients treated with intermediate or therapeutic UFH. TEG parameters and CCTs are analyzed by logistic regression with thrombosis or hemorrhage as dependent variables.

### 1.4. Rationale

Admission levels of biomarkers (e.g., elevated D-dimer) have been identified as prognostic indicators of thrombohemorrhagic events for COVID-19 patients [15,25,26,30,31]. However, current guidelines no longer recommend empiric escalation to intermediate dosing based on these prognostic factors [32,33]. Rather, they indicate the use of therapeutic dose anticoagulation only in the presence of macrovascular thrombosis. Our goal here was to not only identify those patients at risk, but also to prevent these complications in a precision-based and goal-directed fashion using adjunctive TEG. Moreover, it was observed that the hemostatic phenotype of these severely ill patients can rapidly change in a timeframe of hours, or even demonstrate both the thrombotic and hemorrhagic phenotypes simultaneously. Therefore, our multivariate analyses did not center around prognostic indicators at admission; rather, our analyses focused on clinically driven, point-of-care information that required daily, and sometimes multiple times daily, monitoring. From the 79 ICU patients treated only with UFH, we attempted to identify those point-of-care TEG/CCT parameters that would prevent hemorrhage and thrombosis for severely ill COVID-19 patients.

## 2. Materials and Methods

### 2.1. Patient Population and Setting

This study was approved by the institutional review board of Saint Joseph Regional Medical Center (Mishawaka, IN, USA). All patients were aged ≥18 years and diagnosed with SARS-CoV-2 infection by nasopharyngeal/oropharyngeal swab or sputum specimen. Diagnosis of COVID-19 was confirmed by reverse transcriptase-polymerase chain reaction assay based on the World Health Organization (WHO) standard, that targets the SARS-CoV-2 E gene and RdRp gene, or by positive SARS-CoV-2 IgM antibody (BioFire Respiratory 2.1 Panel, BioFire Diagnostics, Salt Lake City, UT, USA) [34].

As mentioned above, this study is an expanded retrospective study during the same timeframe as a previously published pilot study that comprised a heterogeneous group of 31 patients treated with intermediate or therapeutic UFH or enoxaparin from 26 April 2020 to 15 September 2020 [29]. Here, of 531 COVID-19-positive hospital admissions from 26 April 2020 to 1 December 2020, only patients with COVID-19 pneumonitis who were admitted to the ICU and treated with intravenous UFH and had daily CCT and TEG data were included in this analysis.

### 2.2. Unfractionated Heparin Therapy

Prior to 16 September 2020, patients without known macrovascular thrombosis but with elevated D-dimer (>3 fibrinogen equivalent units) and unclear thrombosis risk received the empiric escalation of standard prophylaxis to intermediate dose UFH, which was recommended during this period of the pandemic [15]. At this time, the intermediate dose was defined as 12 units/h/kg infusion. Patients with macrovascular thrombosis received therapeutic bolus UFH also according to the standard pre-pandemic protocol [35]. The therapeutic dose was defined as 60 units/kg bolus followed by 12 units/h/kg infusion.

**Figure 1:**
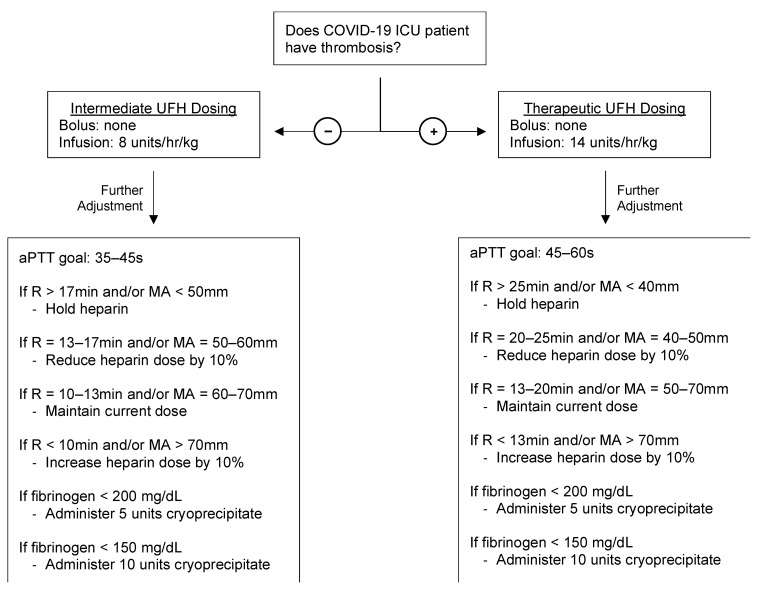
Sample TEG/CCT-based protocol for UFH dosing of hospitalized COVID-19 patients. The presence/absence of a thrombosis determines initial UFH dosing. Further goal-directed titration of UFH was achieved via monitoring of aPTT and TEG parameters R and MA. Adopted from protocols for guiding heparin in ECMO, liver transplantation, cardiac surgery, and trauma surgery [22,36]. aPTT, activated partial thromboplastin time; CCT, conventional coagulation test; COVID-19, coronavirus disease 2019; ICU, intensive care unit; MA, maximum amplitude; R, reaction time; TEG, thromboelastography; UFH, unfractionated heparin.

Due to the observed bleeding rate, from 16 September 2020 to 1 December 2020, patients were selectively administered a non-bolus UFH protocol, individually titrated based on daily monitoring with TEG/CCT [29]. Figure 1 is a sample TEG/CCT algorithm, and in some difficult clinical scenarios wherein patients expressed thrombotic and hemorrhagic phenotypes simultaneously, clinical judgment by the hematologist and transfusion specialist trumped this sample algorithm. Moreover, evidence-based guidelines were actively evolving at this point in the pandemic, and due to expanding indications, remdesivir, dexamethasone, and convalescent plasma were administered with increased frequency during this time period.

### 2.3. Laboratory and Clinical Measures

Data were obtained by the CAC committee in daily review of all admissions to the hospital who received infectious disease consultation. Collected patient data included age, gender, comorbidities, clinical description of bleeding and thrombotic events, laboratory tests, and anticoagulant dosing. PT and aPTT were measured using the Sysmex CA-1500 with additives Innovin and CaCl_2_. Fibrinogen was quantified in samples mixed with thrombin and Owren’s Veronal buffer. D-dimer was measured as fibrinogen equivalent units with the Innovance D-dimer Assay. Platelets were counted with a Sysmex XP-2000 (all from Siemens Medical Solutions, Malvern, PA, USA). TEG tracings were collected using the TEG 5000 hemostasis analyzer with the activator kaolin (Haemonetics, Braintree, MA, USA). TEG parameters include R, k, α-angle, maximum amplitude (MA), and lysis at 30 min (LY30). The CCTs were also measured and recorded at least once daily throughout the patients’ hospitalizations. Anti-Xa levels were not routinely used to monitor hemostasis in COVID-19 patients due to hospital laboratory limitations, as the test was not available during the evenings and weekends and results were not rapidly available in a fashion conducive to quality intensive care.

Bleeding was defined by the American Association of Blood Banks’ Modified WHO Bleeding Scale grade ≥2 (grade 2, mild blood loss; grade 3, gross blood loss that requires transfusion; grade 4, debilitating blood loss causing severe hemodynamic instability [associated with fatality]) [37]. Adjudication of WHO Bleeding Scale grade was agreed upon by two hematologists during retrospective chart review according to patient verbal responses, physical exam findings, and lab results (e.g., acute anemia with heme-positive stool). These hematologists were not blinded to the study design nor the TEG/CCT-based protocol. The WHO Bleeding Scale is a widely used ordinal scale to assess bleeding [38]. However, high inter-observer variability has been reported [39]. Macrovascular thrombosis was classified by radiographic findings or lack of compressibility of proximal veins on B-mode ultrasound [40].

### 2.4. Statistical Analysis

R version 3.6.0. was used for all statistical analysis. Demographics, comorbidities, hematologic events, blood products, interventions, length of stay, and mortality were analyzed for statistical differences via two-sample unpaired *t*-tests and chi-square contingency tests for bleeding versus non-bleeding patients, patients with thrombosis versus patients with no thrombosis, and for the group treated with bolus UFH and no TEG/CCT-algorithm versus the group treated with non-bolus UFH and adjunctive TEG/CCT-algorithm. A value of *p* < 0.05 was the threshold for significance.

Two stepwise multivariate logistic regression analyses were also performed. The first logistic regression used bleeding versus non-bleeding as the dependent variable. The second logistic regression used thrombosis versus no thrombosis as the dependent variable. In both regression analyses, independent variables included TEG parameters R, k, α-angle, and MA and the CCTs PT, aPTT, fibrinogen, platelet count, and D-dimer. The analyzed data included laboratory values on the day of bleeding/thrombosis for the bleeding/thrombotic patients and average values throughout hospitalization for the non-bleeding/non-thrombotic patients. The R^2^ calculated was a McFadden’s Pseudo R^2^.

## 3. Results

### 3.1. Patient Demographics and Clinical Characteristics

This study included 79 COVID-19 patients in the ICU. The median age was 71 (IQR, 16) years and the median BMI was 31.2 (IQR, 7.4) kg/m^2^. Females comprised 27 (34.2%) patients. For the UFH dose at ICU admission, 46 (58.2%) patients received intermediate dose, 16 (20.2%) received bolus therapeutic, and 17 (21.5%) received non-bolus therapeutic. Invasive ventilation for respiratory failure was required in 19 (24.1%) patients. While hospitalized, 15 (19.0%) patients expired. Other relevant comorbidities, hematologic events, length of stay, and blood product administration are detailed in the following sections. TEG parameter LY30 maintained a reading of less than 0.8% for nearly all patients in the study period, and was, thus, not included in the analysis.

### 3.2. Hemorrhagic Complications

Among the 79 patients, 12 (15.2%) demonstrated a clinically significant hemorrhage (Table 1). Among these 12 bleeders, 6 (50%) were administered intermediate UFH at ICU admission, and 6 (50%) were administered bolus therapeutic UFH. The median number of days between admission to the day of bleeding was 10.0 (IQR, 6.3) days. The location of bleeds included 2 (16.7%) gastrointestinal, 1 (8.3%) hemothorax, 3 (25.0%) retroperitoneal, 5 (41.7%) intramuscular, and 1 (8.3%) at vascular access. Compared to non-bleeding patients, there was a significantly greater number of bleeding patients who were administered packed red cells, platelets, and fresh frozen plasma. Five (41.7%) bleeding patients received surgical intervention. The median ICU length of stay for bleeding patients was 25.5 (IQR, 10.5) days, nearly double that of the non-bleeding patients (Table 1).

With hemorrhage as the dependent variable, a stepwise multivariate logistic regression analysis starting with all TEG parameters and CCTs identified a model using R (*p* = 0.016), fibrinogen (*p* = 0.006), D-dimer (*p* = 0.063), and aPTT (*p* = 0.084) with an R^2^ of 0.798 (*p* < 0.001). The adjusted R^2^ is 0.787 (Table 2). Despite D-dimer and aPTT showing statistical insignificance as independent variables, a forced model using only R and fibrinogen had an R^2^ of 0.610 (*p* < 0.001), suggesting an appreciable contribution of D-dimer and aPTT in predicting hemorrhage.

### 3.3. Thrombotic Complications

Among the 79 patients, 20 (25.3%) had an identifiable macrovascular thrombus. Among these 20 thrombotic patients, 9 (45%) were administered intermediate UFH at ICU admission and later developed thrombosis; 5 (25%) were administered bolus therapeutic UFH at ICU admission, and 6 (30%) were administered non-bolus therapeutic UFH. Among the 20 thrombotic patients, there were 32 total clots. Locations included 14 (43.8%) pulmonary emboli, 5 (15.6%) iliac venous system, 4 (12.5%) lower extremity deep veins, 3 (9.8%) internal jugular veins, 2 (6.3%) upper extremity deep veins, 2 (6.3%) renal vein, 1 (3.1%) inferior vena cava, and 1 (3.1%) renal artery. Compared to non-thrombotic patients, there was a significantly greater number of thrombotic patients who required surgical intervention. Seven (35%) thrombotic patients received surgical intervention. Between the thrombotic and non-thrombotic patients, there was no significant difference in demographics, comorbidities, UFH dose, blood product use, invasive ventilation, length of stay, or mortality (Table 3).

With thrombosis as the dependent variable, a stepwise multivariate logistic regression analysis starting with all TEG parameters and CCTs identified a model using D-dimer (*p* < 0.001), R (*p* = 0.002), MA (*p* < 0.001), and α-angle (*p* = 0.014) with an R^2^ of 0.381 (*p* < 0.001) The adjusted R^2^ is 0.348 (Table 2).

### 3.4. Bolus versus Non-Bolus UFH with Adjunctive TEG/CCT Algorithm

Among the 12 patients who bled, 11 (91.6%) patients bled prior to using the non-bolus TEG/CCT-based protocol, whereas only 1 (8.3%) patient bled after adopting the protocol. After establishing the non-bolus TEG/CCT-based protocol, there was a significant decrease in bleeding events (*p* < 0.001) and the number of patients administered packed red blood cells (*p* = 0.031) and fresh frozen plasma (*p* < 0.001). Between the two groups, there was no significant difference in the demographics, comorbidities, thrombotic events, interventions, length of stay, or mortality rate (Table 4).

## 4. Discussion

This single center, retrospective study corroborates existing literature regarding the high incidence of thrombohemorrhagic complications in severely ill COVID-19 patients [2,9,12,14,15,25,30,31,41,42]. Early in the pandemic, some reports demonstrated high bleeding rates similar to our early findings [12,14,15,25]. One observational study demonstrated a bleeding rate as high as 21% for critically ill patients [9]. Larger, recent trials have reported bleeding incidences ranging from 2.5% to 8%, varying with disposition, anticoagulant dose, and the hemorrhage classification used [17,30,41,42].

However, this study includes patients treated at a time in the pandemic when evidence was actively evolving, and empiric escalation of the anticoagulant dose was recommended for those patients with elevated D-dimer at admission and without macrovascular thrombosis [15]. Interestingly, we observed six bleeding events with intermediate UFH and six bleeding events with bolus therapeutic UFH. No bleeding events were observed in patients administered non-bolus therapeutic UFH, although this finding may be attributable to the gained experience of the CAC committee in anticoagulating these patients in the latter half of this study. Among these three dosages, none of them appeared to prevent VTEs during hospitalization, which corroborates the recent findings of the ACTION trial [42]. Another significant finding among our bleeding patients was that their median length of ICU stay was nearly double that of the patients that did not suffer a bleeding event. This may suggest two things: First, patients who bleed are among the sickest and will require the most intensive care; second, diligent care to prevent hemorrhage in COVID-19 patients is important to decrease hospital and healthcare system costs. Patients with hemorrhagic events required more blood product administration and surgical intervention compared to the non-hemorrhagic group. The thrombotic group also demonstrated a higher need for surgical intervention. In contrast, however, the thrombotic group did not have a greater length of stay compared to those patients without thrombosis.

The logistic regression model for hemorrhage identified increased R, decreased fibrinogen, decreased D-dimer, and increased aPTT as the important variables for predicting hemorrhage with an R^2^ of 0.798. Compared to our previous pilot study of 31 heterogeneous patients treated with enoxaparin and UFH, R time, aPTT, and fibrinogen were also shown here to be significant on the day of bleeding among 79 patients treated only with UFH [29]. The positive correlation of prolonged R time with bleeding was anticipated because, through its indirect effect on thrombin formation via antithrombin, UFH primarily affects the time to initial thrombin formation and subsequent fibrin formation [43]. The negative correlation between fibrinogen and bleeding found in the logistic regression model likely relates to the pathophysiology of CAC. Hepatic synthesis of fibrinogen has been shown to increase two- to ten-fold as an acute phase reactant during acute inflammation [44]. Moreover, the early hypercoagulable state of CAC is associated with increased fibrinogen and D-dimer, and fibrinolytic shutdown has been demonstrated on TEG [45,46,47,48]. The hemorrhagic phenotype of CAC may correspond to the later reduction in fibrinogen synthesis as the acute inflammatory response abates, coupled with the rebalancing of the fibrinolytic system [10,16,26,46]. Fibrin-thrombin complexes protect thrombin from inactivation by antithrombin and have been shown to require twenty times more heparin for inactivation when compared to free thrombin [43]. As the fibrinolytic shutdown state of CAC dissipates and fibrinogen levels begin to decrease, more free thrombin may become accessible for inactivation by antithrombin. In addition, low levels of antithrombin have been demonstrated in hospitalized COVID-19 patients upon admission. Because these levels also return to normal with the dissipation of the acute inflammatory response, these patients would be expected to exhibit increased heparin sensitivity [16,26,47,48]. We hypothesize that these combined hemostatic derangements in fibrinogen and antithrombin levels may in part explain the late development of increased heparin sensitivity and resulting hemorrhage demonstrated in some hospitalized COVID-19 patients treated with heparinoids.

The logistic regression model for thrombosis identified increased D-dimer, decreased R, increased MA, and decreased α-angle as the important variables for predicting thrombosis with an R^2^ of 0.381. This model demonstrates weak predictability of thrombosis; in previous studies, thromboelastography parameters have not shown to be reliable predictors of thrombosis in COVID-19 patients [25,28]. High platelet-fibrin interaction strength, associated with increased MA, has been replicated in a few other studies for patients with CAC [28,49,50,51]. A short R has been demonstrated less consistently [25,28,49,51]. The significance of decreased α-angle in this model may allude to the weak predictability for thrombosis, as hypercoagulable states more consistently demonstrate increased α-angle. D-dimer has been a reliable predictor for thrombosis, and our results corroborate the existing literature [15,25,30,31].

Recently, an international, multi-platform randomized controlled trial (mpRCT) was initiated to elucidate the risks and benefits of full therapeutic anticoagulation for hospitalized COVID-19 patients. This tri-platform trial comprised the Randomized, Embedded, Multi-factorial Adaptive Platform Trial for Community-Acquired Pneumonia (REMAP-CAP), Accelerating COVID-19 Therapeutic Interventions and Vaccines-4 Antithrombotics (ACTIV-4A), and Antithrombotic Therapy to Ameliorate Complications of COVID-19 (ATTACC) studies [52,53]. The interim analysis released on 22 January 2021 demonstrated that early therapeutic anticoagulation in moderately ill non-ICU patients decreased rates of mechanical ventilation and mortality, but may be associated with bleeding [52]. These large, heterogeneous trials—comprising over 4000 patients across five countries and 30 hospitals—suggest that severely ill ICU patients may not benefit from full therapeutic anticoagulation unless macrovascular thromboses are present. The three studies of the mpRCT were harmonized for patients to receive standard dosing of UFH or low-molecular-weight heparin. However, none of these three studies mention monitoring anticoagulation with adjunctive viscoelastic testing nor with CCTs. The monitoring of anticoagulation for these patients is not well-defined or pre-specified and remains an important, yet difficult, challenge.

In our single-center study, patients with and without macrovascular thrombosis were treated with UFH before and after developing a TEG/CCT-based protocol. Our analysis corroborates the interim findings of the mpRCT, demonstrating that full therapeutic anticoagulation may be harmful [17,52]. In the mpRCT, ICU level of care was defined as any patient who required high flow nasal oxygen, invasive or noninvasive mechanical ventilation, vasopressor support, or ECMO [16]. The risks of bleeding are far greater in the sicker, mechanically ventilated, and ECMO populations. Benefit may be procured with therapeutic anticoagulation for those patients in the ICU who are less ill, as shown by the mortality benefit with therapeutic anticoagulation in the moderately ill group of the mpRCT [16,22]. It is possible that the mortality for therapeutic anticoagulation in the severely ill ICU group of the mpRCT was confounded by the inclusion of mechanically ventilated and ECMO patients in the same group as those less ill who only required high flow nasal oxygen or noninvasive ventilation. Recently, however, the INSPIRATION trial tested empiric intermediate versus standard prophylactic anticoagulation for ICU patients without macrovascular thrombosis [41]. The trial demonstrated no significant difference in the incidence of thrombosis, escalation to ECMO therapy, or 30-day mortality between the two treatment groups, further corroborating the ACTIV-4A findings of futility with empiric dose escalation.

The severely ill COVID-19 patient in the ICU requires daily monitoring of hemostasis as the intensity of the cytokine storm decreases and the patient transitions from heparinoid resistance to heparinoid hypersensitivity. It is unknown whether viscoelastic testing or a specific protocol was used in managing anticoagulation among the many hospitals in the mpRCT. Our analysis suggests that adjunctive TEG enables anticoagulation of the severely ill ICU patient without the hemorrhagic complications encountered by these larger studies where the protocols did not call for such intense monitoring and personalization of dosing. Here, the results indicate that adjunctive TEG-guided goal-directed therapy of UFH reduced bleeding events for those treated with intermediate or therapeutic anticoagulation. Our single-center, observational demonstration of intermediate or therapeutic UFH guided by adjunctive TEG may justify this precision-based medicine approach to providing safer anticoagulation to severely ill COVID-19 patients [4,20].

Since recent studies of therapeutic anticoagulation for COVID-19 patients have demonstrated the narrow therapeutic window of heparinoids, we hypothesized that adjunctive TEG may provide safer, goal-directed UFH titration. A similar rationale has been cited for managing the spectrum of coagulopathies occurring in patients on ECMO who require carefully personalized titration not only with aPTT, but also with TEG and anti-Xa levels [22,23]. In spite of decades-long anticoagulation for ECMO patients “with little guidance regarding which laboratory test to monitor heparin, many institutions have turned to literature and experience to develop their own heparin protocol for ECMO” [22]. This has led many institutions to adopt adjunctive TEG to assist in the guidance of anticoagulation for the ECMO patient. During this historically unique pandemic, we elected to follow hematologists’ and clinicians’ examples with ECMO. Therefore, we adopted a similar strategy of aPTT monitoring every six hours of UFH therapy with at least once daily D-dimer, fibrinogen, platelet count, and adjunctive TEG analysis guided by coagulation specialists. Like an ECMO protocol, anticoagulation was guided by these daily laboratory measurements under the auspices of a hematologist or transfusion specialist-led coagulation committee. Significantly, the application of an ECMO-like anticoagulation protocol for COVID-19 patients not only decreased bleeding events in our study, but also decreased transfusion requirements in the form of packed red blood cells and fresh frozen plasma. There was no statistically significant difference in surgical interventions, invasive ventilation, length of stay, or mortality after adopting the TEG/CCT non-bolus protocol.

A limitation of this study is the relatively small sample size of 79 COVID-19 ICU patients. However, when compared to many of the papers that have been published focusing on TEG or other viscoelastic tests in the COVID-19 population, this is a relatively high number of patients [28]. Such small observational studies, in a period of sparse evidence-based medicine, have been commented on by authoritative clinicians and these studies provide the foundation for future RCTs [24]. Another limitation is the unblinded observational study design wherein the second cohort received anticoagulation months into the pandemic when the CAC committee had more clinical experience in anticoagulating these patients, as well as evolved treatment guidelines. Moreover, anti-Xa levels were not used to guide UFH therapy in this study. At our hospital and at many hospitals in the United States, around the clock availability of the anti-Xa assay is lacking. The test is performed only during weekdays at our medical center, rendering this assay of limited utility when immediate intensive care management of UFH is required. Turnaround time of up to six hours during daytime hours does not permit the timely and effective adjustment of anticoagulation necessary for these patients. The benefit of monitoring continuous UFH therapy with either aPTT or anti-Xa is based on evolving evidence [35].

## 5. Conclusions

Like ECMO, an institution-specific titration strategy for anticoagulating COVID-19 patients may provide safer and more effective therapy. Here, we demonstrated that adjunctive TEG may have decreased bleeding rates but did not decrease thrombosis rates in the ICU. Significantly, we developed a model for hemorrhage on the day of bleeding wherein increased R time, decreased fibrinogen, decreased D-dimer, and increased aPTT were predictive of bleeding.

## Figures and Tables

**Table 1:** Clinical characteristics of 12 bleeding and 67 non-bleeding COVID-19 patients in the ICU. Baseline anticoagulant dose indicates the unfractionated dose administered upon ICU admission. WHO Bleeding Scale grade of 2 indicates mild blood loss, grade 3 indicates gross blood loss requiring transfusion, and grade 4 indicates debilitating blood loss [37]. The number of days to bleed is measured from hospital admission. Statistical significance is indicated by an asterisk (*). BMI, body mass index; COPD, chronic obstructive pulmonary disease; COVID-19, coronavirus disease 2019; ICU, intensive care unit; IQR, interquartile range; WHO, World Health Organization.

	Bleed (*n* = 12)	No Bleed (*n* = 67)	*p*-Value
Age (years), median (IQR)	63.5 (17.8)	73.0 (15.5)	0.065
Female, *n* (%)	6 (50.0%)	21 (31.3%)	0.210
BMI (kg/m^2^), median (IQR)	33.7 (7.0)	29.9 (6.6)	0.135
Comorbidities, *n* (%)			
COPD	3 (25.0%)	10 (14.9%)	0.386
Tobacco use	2 (16.7%)	10 (14.9%)	0.877
Coronary artery disease	1 (8.3%)	12 (17.9%)	0.410
Heart failure	3 (25.0%)	5 (7.5%)	0.064
Hypertension	10 (83.3%)	44 (65.7%)	0.226
Type II diabetes mellitus	5 (41.7%)	28 (41.8%)	0.994
Renal failure	4 (33.3%)	9 (13.4%)	0.087
Baseline anticoagulant dose, *n* (%)			
Intermediate	6 (50.0%)	40 (59.7%)	0.530
Bolus therapeutic	6 (50.0%)	10 (14.9%)	0.005 *
Non-bolus therapeutic	0 (0.0%)	17 (25.4%)	0.049 *
Bleeding complications, *n* (%)			
Gastrointestinal	2 (16.7%)	-	-
Hemothorax	1 (8.3%)	-	-
Retroperitoneal	3 (25.0%)	-	-
Intramuscular	5 (41.7%)	-	-
Vascular access	1 (8.3%)	-	-
WHO bleeding scale score, *n* (%)			
Grade 2	3 (25.0%)	-	-
Grade 3	8 (66.7%)	-	-
Grade 4	1 (8.3%)	-	-
Time to bleed (days), median (IQR)	10.0 (6.3)	-	-
Blood Products, *n* (%)			
Packed red cells	6 (50.0%)	14 (20.9%)	0.033 *
Cryoprecipitate	7 (58.3%)	23 (34.3%)	0.115
Platelets	4 (33.3%)	2 (3.0%)	<0.001 *
Fresh frozen plasma	10 (83.3%)	19 (28.4%)	<0.001 *
Interventions, *n* (%)			
Surgical	5 (41.7%)	9 (13.4%)	0.018 *
Invasive ventilation	5 (41.7%)	14 (20.9%)	0.121
Length of ICU stay (days), median (IQR)	25.5 (10.5)	13.0 (12.0)	0.005 *
Mortality, *n* (%)	3 (25.0%)	12 (17.9%)	0.564

**Table 2:** Multivariate logistic regression models for bleeding and thrombosis. aPTT, activated partial thromboplastin time; MA, maximum amplitude; R, reaction time.

Dependent Variable	Parameter	Coefficient	*p*-Value	R^2^	Adj. R^2^
Bleeding	Intercept	−2.509	0.374	0.798	0.787
	R	0.507	0.016		
	Fibrinogen	−0.039	0.006		
	D-dimer	−0.441	0.063		
	aPTT	0.126	0.084		
Thrombosis	Intercept	−2.436	0.467	0.381	0.348
	D-dimer	0.195	<0.001		
	R	−0.545	0.002		
	MA	0.220	<0.001		
	α-angle	−0.152	0.014		

**Table 3:** Clinical characteristics of 20 thrombotic patients and 59 non-thrombotic COVID-19 patients in the ICU. Baseline anticoagulant dose indicates the unfractionated dose administered upon ICU admission. Statistical significance is indicated by an asterisk (*). BMI, body mass index; COPD, chronic obstructive pulmonary disease; COVID-19, coronavirus disease 2019; DVT, deep vein thrombosis; IVC, inferior vena cava; ICU, intensive care unit; IQR, interquartile range; LE, lower extremity; UE, upper extremity.

	Thrombosis (*n* = 20)	No Thrombosis (*n* = 59)	*p*-Value
Age (years), median (IQR)	67.0 (21.0)	73.0 (15.0)	0.362
Female, *n* (%)	9 (45.0%)	18 (30.5%)	0.238
BMI (kg/m^2^), median (IQR)	31.8 (5.8)	31.2 (7.3)	0.628
Comorbidities, *n* (%)			
COPD	2 (10.0%)	11 (18.6%)	0.368
Tobacco use	2 (10.0%)	10 (16.9%)	0.454
Coronary artery disease	2 (10.0%)	11 (18.6%)	0.368
Heart failure	2 (10.0%)	6 (10.2%)	0.983
Hypertension	11 (55.0%)	43 (72.9%)	0.137
Type II diabetes mellitus	11 (55.0%)	22 (37.3%)	0.165
Renal failure	4 (20.0%)	9 (15.3%)	0.621
Baseline anticoagulant dose, *n* (%)			
Intermediate	9 (45.0%)	37 (62.7%)	0.165
Bolus therapeutic	5 (25.0%)	11 (18.6%)	0.541
Non-bolus therapeutic	6 (30.0%)	11 (18.6%)	0.286
Localization of clots (*n* = 32), *n* (%)			
Pulmonary embolus	14 (43.8%)	-	-
Iliac DVT	5 (15.6%)	-	-
LE DVT	4 (12.5%)	-	-
Internal jugular	3 (9.8%)	-	-
UE DVT	2 (6.3%)	-	-
Renal vein	2 (6.3%)	-	-
IVC	1 (3.1%)	-	-
Renal artery	1 (3.1%)	-	-
Blood Products, *n* (%)			
Packed red cells	4 (20.0%)	16 (27.1%)	0.527
Cryoprecipitate	5 (25.0%)	25 (42.4%)	0.167
Platelets	1 (5.0%)	5 (8.5%)	0.612
Fresh frozen plasma	6 (30.0%)	23 (39.0%)	0.471
Interventions, *n* (%)			
Surgical	7 (35.0%)	7 (11.9%)	0.019 *
Invasive ventilation	4 (20.0%)	15 (25.4%)	0.624
Length of ICU stay (days), median (IQR)	11.0 (19.0)	14.0 (12.0)	0.964
Mortality, *n* (%)	2 (10.0%)	13 (22.0%)	0.236

**Table 4:** Clinical characteristics before (1st Group) and after (2nd Group) establishment of the adjunctive TEG/CCT-based protocol to guide unfractionated heparin dosing. Statistical significance is indicated by an asterisk (*). BMI, body mass index; CCT, conventional coagulation test; COPD, chronic obstructive pulmonary disease; IQR, interquartile range; VTE, venous thromboembolism.

	1st Group (*n* = 35)	2nd Group (*n* = 44)	*p*-Value
Age (years), median (IQR)	70.0 (19.5)	74.5 (16.0)	0.123
Female, *n* (%)	11 (31.4%)	16 (36.4%)	0.646
BMI (kg/m^2^), median (IQR)	31.6 (6.7)	30.8 (7.7)	0.340
Comorbidities, *n* (%)			
COPD	7 (20.0%)	6 (13.6%)	0.449
Tobacco use	6 (17.1%)	6 (13.6%)	0.666
Coronary artery disease	6 (17.1%)	7 (15.9%)	0.883
Heart failure	5 (14.3%)	3 (6.8%)	0.274
Hypertension	26 (74.3%)	28 (63.6%)	0.312
Type II diabetes mellitus	15 (42.9%)	18 (40.9%)	0.862
Renal failure	4 (11.4%)	9 (20.5%)	0.283
Hematologic Events, *n* (%)			
Bleed during hospitalization	11 (31.4%)	1 (2.3%)	<0.001 *
VTE during hospitalization	8 (22.9%)	12 (27.3%)	0.654
Blood Products, *n* (%)			
Packed red cells	13 (37.1%)	7 (15.9%)	0.031 *
Cryoprecipitate	13 (37.1%)	17 (38.6%)	0.892
Platelets	4 (11.4%)	2 (4.5%)	0.251
Fresh frozen plasma	24 (68.6%)	5 (11.4%)	<0.001 *
Interventions, *n* (%)			
Surgical	8 (22.9%)	6 (13.6%)	0.286
Invasive ventilation	9 (25.7%)	10 (22.7%)	0.758
Length of stay (days), median (IQR)	18.0 (13.5)	11.0 (13.3)	0.133
Mortality, *n* (%)	5 (14.3%)	10 (22.7%)	0.342

## Data Availability

The data presented in this study are available on request from the corresponding author.
